# A Method for Correction of Dynamic Errors When Measuring Flat Surfaces

**DOI:** 10.3390/s24165154

**Published:** 2024-08-09

**Authors:** Dimitar Dichev, Dimitar Diakov, Iliya Zhelezarov, Stefan Valkov, Maria Ormanova, Ralitza Dicheva, Oleksandr Kupriyanov

**Affiliations:** 1 Department of Machine and Precision Engineering, Technical University of Gabrovo, 4 H. Dimitar Str., 5300 Gabrovo, Bulgaria; dichevd@abv.bg (D.D.); izhel@tugab.bg (I.Z.); 2 Department of Precision Engineering and Measurement Instruments, Technical University of Sofia, 1756 Sofia, Bulgaria; diakov@tu-sofia.bg (D.D.); ralitsa.dichevaa@gmail.com (R.D.); 3 Institute of Electronics, Bulgarian Academy of Sciences, 72 Tzarigradsko Chaussee Blvd., 1784 Sofia, Bulgaria; maria.ormanova87@gmail.com; 4 Department of Mathematics, Informatics and Natural Sciences, Technical University of Gabrovo, 4 H. Dimitar Str., 5300 Gabrovo, Bulgaria; 5 Department of Information Computer Technologies and Mathematics, Ukrainian Engineering Pedagogics Academy, 16 Universitetskaya St., 61003 Kharkov, Ukraine; a_kupriyanov@uipa.edu.ua

**Keywords:** dynamic error correction, planar surface measurement, Kalman filter, measurement accuracy

## Abstract

This paper presents a new method for correction of dynamic errors occurring when measuring flat surfaces in the presence of mechanical effects. Mechanical effects cause inertial forces and moments that affect the moving components of measuring instruments, thereby causing dynamic errors. The study proposes a mathematical model, on the basis of which algorithms for correction of dynamic errors can be developed. The basic concept of the model is based on determining the optimal estimate in the current coordinate point on the basis of the theoretical model of the measured surface and the information from the measurement that contains errors caused by internal and external factors. Based on this model, an algorithm for real-time data processing has been developed. The algorithm works in “predictor-corrector” mode at each step of which the best estimate is obtained. The estimate is based on minimizing the variance of a random component in which the main values are formed from the accumulated statistical data of the error of the model and the measurement error. This paper presents the results of experimental studies, carried out with simulations of mechanical effects in four modes. The results confirm the high efficiency of the algorithm for high-accuracy measurement of flat surfaces in the presence of mechanical effects.

## 1. Introduction

The constant increase in the quality requirements of machine-building products leads to the need for higher accuracy of technological and measuring equipment, as well as for constant monitoring of their condition throughout their entire life cycle [1]. Machine tools, as the main equipment in machine-building enterprises, are key to the production of high-quality elements. The accuracy of the manufactured parts depends on the correct geometric shape of the supporting surfaces of the machines [2,3].

Coordinate measuring machines (CMM) are widely used in production, and the deviation from the flatness of the working and measuring surfaces is an important parameter for their accuracy [4]. Effective measurement of plane surfaces requires high accuracy and reliability of results, even in the presence of various external influences such as vibration, electromagnetic noise, and temperature deformations.

Mechanical external effects, such as vibrations and shocks, cause inertial forces and moments that affect the moving elements of the measuring instruments, leading to additional dynamic errors [5].

Dynamic errors can be structured in the general metrological concept of the theory of errors; however, the methods for accomplishing the tasks related to the analysis, experimental study, and development of methods for their elimination are specific [6,7]. For example, in [8] a method for dynamic optimization based on the developed dynamic model of the probe of CMM is proposed. The error model in [9] is built with the recursive least squares (RLS) identification technique by taking probing acceleration and the six geometric errors, derived from the position of the tip of the probe. An adaptive method with active control of the process of machining parts is proposed in [10]. In this study, the positioning errors of the tool are modelled by approximating the components of the dynamic error as polynomial functions.

These examples show that the operation of the measuring instruments is characterized not only by the external inertial effects, but also by the internal effects. The internal inertial effects (as well as the external ones) cause dynamic errors that in most cases cannot be ignored, and the operation mode of these measuring instruments and systems can be categorized as dynamic [11,12,13]. The dynamic mode of operation differs from the static one, and the analysis of accuracy in this mode of operation poses additional conditions that must be considered when conducting this study [14]. These conditions are determined primarily by the characteristics of the processes influencing the formation of dynamic errors. In this regard, two main concepts are observed in the analysis of dynamic errors and the optimization of accuracy characteristics of instruments for measuring linear quantities in the dynamic mode of operation. The first concept states that the developed theoretical models for investigation and optimization are based on methods that allow specific dynamic characteristics of input-output processes to be studied in accordance with the logic and metrological theory of static measurements. In this regard, the most commonly used methods are neural networks [15,16,17,18]; the least squares [9,19]; Monte Carlo [20]; and statistical models for analysis [21,22,23,24]. The basis for the second concept is the increasing use of methods based on the information from external sources. Nowadays, this tendency is intensified due to the ever-increasing quality of MEMS sensors and sensor systems, allowing active compensation of dynamic errors [25]. MEMS sensors offer significant advantages such as compactness, low cost, and high sensitivity [26]. However, they also have drawbacks, such as limited accuracy in the presence of noise and temperature changes. The introduction of MEMS systems in measurement processes requires compliance with these limitations and the development of algorithms for their correction.

The published results are related to this tendency and are based on the information from external sources such as laser interferometric systems. The excellent capabilities of these systems for building external reference elements related to the geometry of technological machines and measuring systems create good prospects for developing methods for estimating the components of the error under different dynamic conditions [20,25,27,28,29,30]. An original approach for analysis and research through external reference elements is proposed in sources [31,32,33,34], where methods for study and the identification of the main components of dynamic errors are developed on the basis of artefacts with different geometries.

The studies in most publications mentioned above are focused mainly on the analysis of the characteristics and compensation of dynamic errors caused by internal forces and moments. Limiting research to only specific sources of dynamic errors does not allow the development of algorithms and procedures for improving measurement accuracy that can be efficient in a wide range of internal and external effects. Further problems in measuring flat surfaces arise from the need to carry out a great number of measurements, and subsequently to a significant amount of work related to the mathematical processing of the results. In this case, the measurement results should be obtained as soon as possible in order to reduce the downtime of processing equipment.

Based on all that has been said so far, the following requirements that need to be met by high-performance and high-quality systems for measuring flat surfaces can be:

- accuracy;
- invariance of measurement results to input data errors;
- quick measurement process;
- quick processing algorithms;
- effective use in different production environments;
- universality of algorithms for correction of dynamic errors regarding the type of the measuring systems and the nature of the inertial effects.

It is a challenging task to fulfil all these requirements in one common measurement concept. One of the most promising methods for solving this problem is to develop models and algorithms for active compensation of dynamic errors, where the obtained optimal estimate meets the criterion of minimum variance of the model and measurement errors.

## 2. A Mathematical Model of the Data Processing Algorithm

Instruments for measuring the geometry of flat surfaces are designed to work in static mode and are therefore not able to measure the dynamics of quantities. This significantly complicates the analysis and study of the input-output processes in measuring instruments in the conditions of theory of dynamic measurements, where the main parameter is the time component. However, the development of sufficiently accurate and efficient algorithms to compensate for the influence of external effects on the accuracy of measurements of flat surfaces can be done using a combined approach, where the models used in dynamic metrology are adapted to the metrological characteristics of static measurements.

It is appropriate, in this case, to use the well-known system of equations [35,36], which characterises the state of a dynamic system:

(1) $$\begin{array}{c} \dot{s}\left(t\right)=F_{t}\cdot s\left(t\right)+B_{t}\cdot u\left(t\right)+\omega \left(t\right)\\ c\left(t\right)=H_{t}\cdot s\left(t\right)+\upsilon \left(t\right)\; \end{array}$$

where $s\left(t\right)$—vector, defines the state of the dynamic system; $F_{t}$—matrix, determines the dynamic model of the system; $B_{t}$—matrix, determines the extent to which the input effect is rendered to the state vector; $u\left(t\right)$—control effect; $\omega \left(t\right)$—vector, defines the values of the noise in the state vector; $c\left(t\right)$—vector of measurements; $H_{t}$—matrix, defines the relationship between the measurements and the state of the system; $\upsilon \left(t\right)$—vector, defines the values of the noise in the vector of measurements.

The noise $\omega \left(t\right)$ in (1) is a random variable that describes the external and internal disturbances affecting the dynamical system. In the context of the dynamical model (1), we assume that $\omega \left(t\right)$ is a Gaussian distributed random variable with zero mathematical expectation, i.e., $M\left[\omega \left(t\right)\right]=0$ and a covariance matrix describing the variance of the noise, which can be expressed as a diagonal matrix with elements that represent the variances of the corresponding noise components.

The difficulties in compiling a model based on Equation (1) that can be used to compensate for the dynamic errors in measurements where the results are static values are due to two main factors. The first factor is that the measurement results in static mode do not depend on the time component t, which, however, is the main argument in Equation (1). The second factor is due to one important characteristic feature of dynamic systems, according to which the state of the system at a fixed moment of time will depend not only on the values of the studied quantity at this very moment, but also on its values in previous moments of time. It is this characteristic that is the “connecting link” and the logic behind the processes defining the model (1).

Despite these difficulties, there are enough techniques based on metrological principles in the field of static measurements that can be used in this case. For example, the theoretical model of the measured surface can be used as a “connecting” link in the developed method. On the one hand, this model specifies the reference element in measurement procedures covering current, previous, and subsequent coordinate sequences of the measured object, and on the other hand, its mathematical formulation does not contain as an argument the time t. In accordance with all this, the following mathematical model was used [37,38]:

(2) $$\begin{array}{r} z_{k+1}=z_{k}+\Delta z_{k}+\varepsilon_{k}\\ q_{k}=z_{k}+\rho_{k} \end{array}$$

where $z_{k}$—the true value of the linear quantity on the z coordinate at coordinate point $x_{k}\left(k=1,\ldots m\right)$ and $y_{j}\left(j=1,\ldots n\right)$; $\Delta z_{k}$—value, determined by the theoretical model of the measured surface as the difference in the value z in the previous $\left(x_{k}y_{j}\right)$ and current points $\left(x_{k}+1y_{j}\right)$ of the coordinate sequence; $q_{k}$—value, obtained from measurement along the z coordinate at a coordinate point $x_{k}y_{j}$; $\varepsilon_{k}$—random variable, characterizes the model error; $\rho_{k}$—random variable, characterizes the measurement error.

It should be noted that the mathematical model presented in this part of the work is based on the concepts used in the Kalman filter [37,38]. The Kalman filter algorithm is widely used in applications for assessing the state of dynamic systems. The model used in this work is a simplified case of the general algorithm adapted to improve measurements of plane surfaces by compensating for dynamic errors. Special attention is paid to adapting the filter to the specifics of static measurements, and a specific formulation is introduced for processing measurements and predictive estimates. In this context, the work in this part does not aim to present a new model but rather to adapt the existing Kalman algorithm to specific metrological conditions where the time component is not the leading one.

Adapting the Kalman algorithm to the needs of measuring plane surfaces requires special attention to specific measurement conditions where the time component is not a major factor. In classical applications of Kalman’s algorithm, the temporal aspect is important, but in the case of measuring plane surfaces, it is necessary to focus on spatial dependencies and features. Therefore, model (2) has been adapted to take into account both internal and external interference by including the $\varepsilon_{k}$ errors and noise $\omega \left(t\right)$. This adaptation allows for achieving high accuracy in estimating the measured quantities, even in the absence of temporal dynamic changes. On the other hand, in Equation (2), the errors $\varepsilon_{k}$ are random variables characterizing the deviations of the theoretical model from the actual conditions. We assume that $\varepsilon_{k}$ is also a Gaussian-distributed random variable with zero mathematical expectation and variance $\sigma_{\varepsilon_{k}}^{2}$. The covariance structure of these errors is described by a diagonal matrix since it is assumed that the errors are independent and identically distributed.

The first and the second equation of the system (2) define the change, respectively, of the theoretical model and the measurement in the coordinate sequence that is accepted as a measurement structure. The system (2) is in the basis of the concept adopted in this study, according to which the estimate of the geometric quantity at the current coordinate point $x_{k}y_{j}$ can be defined with high accuracy by the model of the geometric object and the information from the measurement that contains errors caused by external and internal effects.

It should be noted that an important condition for the proper functioning of the model set by the system (2) is the precise definition of the errors $\varepsilon_{k}$ and $\rho_{k}$. These characteristics have been determined in accordance with the conditions of the basic principle that is to be achieved as an objective of the operation of the model (2). The objective, according to this principle, is to obtain the closest value to the real coordinate z at the point $x_{k}+1y_{j}$, and not to filter the measurement values with a view to their “smoothing”. With this in mind, the errors of the model $\varepsilon_{k}$ and the measurement errors $\rho_{k}$ are considered to be random variables that do not depend on time. In addition, each of the errors $\varepsilon_{k}$ and $\rho_{k}$ is defined as an independent random variable, i.e., $M\left[\varepsilon_{k}\varepsilon_{k+1}\right]=M\left[\varepsilon_{k}\right]M\left[\varepsilon_{k+1}\right]$ and $M\left[\rho_{k}\rho_{k+1}\right]=M\left[\rho_{k}\rho_{k+1}\right]$. M denotes the mathematical expectation, which is assumed to be zero, i.e., $M\left[\varepsilon_{k}\right]=M\left[\rho_{k}\right]=0$.

The system of Equation (2) makes it possible to develop algorithms on the basis of which to obtain the estimates $z_{k+1}^{0}$ that have the property of optimality with regard to the closest value to the real coordinate z. Determination of the estimates $z_{k+1}^{0}$ can be done by finding a weighting factor $\lambda_{k+1}$ in each step of the iterative process, which gives the best approximation to the real coordinate z based on the inaccurate measurement and the estimate in the step k+1. Subsequently, the optimal estimate at the point $x_{k}+1y_{j}$ will be:

(3) $$z_{k+1}^{0}=\lambda_{k+1}q_{k+1}+\left(1-\lambda_{k+1}\right)\left(z_{k}^{0}+\Delta z_{k}\right)$$

where $q_{k+1}$ is the value obtained from the measurement at the point $x_{k}+1y_{j}$; is the estimate for the k+1st step of the iterative process obtained on the basis of the theoretical model.

The value of the coefficient is $\lambda_{k+1}$ is determined based on minimizing the error $\delta_{k+1}$, which is defined by the difference between the true $z_{k+1}$ and the optimal value $z_{k+1}^{o}$ of the desired quantity, i.e.:

(4) $$\delta_{k+1}=z_{k+1}-z_{k+1}^{0}$$

The relationships between variables known in the literature lead to the following expression [37,38]:

(5) $$\delta_{k+1}=\left(1-\lambda_{k+1}\right)\left(\delta_{k}+\varepsilon_{k}\right)-\lambda_{k+1}\rho_{k+1}.$$

The error δ is a random variable, the probabilistic characteristics of which depend on the adopted measurement procedures, as can be seen from (5). It is necessary to find such a value of $\lambda_{k+1}$ for which the error $\delta_{k+1}$ will have a minimum in each successive step k+1. For this purpose, the minimum of mean squared error is usually used, i.e.:

(6) $$M\left[\delta_{k+1}^{2}\right]\to min$$

The mean squared error can be determined on the basis of (5):

(7) $$M\left[\delta_{k+1}^{2}\right]=\lambda_{k+1}^{2}A-2\lambda_{k+1}B+C$$

where

(8) $$A=M\left[\delta_{k}^{2}\right]+2M\left[\delta_{k}\varepsilon_{k}\right]+2M\left[\delta_{k}\rho_{k+1}\right]+\sigma_{\varepsilon_{k}}^{2}+2M\left[\varepsilon_{k}\rho_{k+1}\right]+\sigma_{\rho_{k+1}}^{2}\;B=M\left[\delta_{k}^{2}\right]+2M\left[\delta_{k}\varepsilon_{k}\right]+M\left[\delta_{k}\rho_{k+1}\right]+\sigma_{\varepsilon_{k}}^{2}+M\left[\varepsilon_{k}\rho_{k+1}\right]\;C=M\left[\delta_{k}^{2}\right]+2M\left[\delta_{k}\varepsilon_{k}\right]+\sigma_{\varepsilon_{k}}^{2}$$

where $\sigma_{\varepsilon_{k}}^{2}=M\left[\varepsilon_{k}^{2}\right]$—variance of the error ε obtained in step k of the iterative process; $\sigma_{\rho_{k+1}}^{2}=M\left[\rho_{k+1}^{2}\right]$—variance of the error, obtained in step k+1 of the iterative process.

Since the errors ε and ρ are independent random variables and the mathematical expectations $M\left[\varepsilon_{k}\right]=M\left[\rho_{k}\right]=0$, it follows that $M\left[\delta_{k}\varepsilon_{k}\right]=M\left[\delta_{k}\rho_{k+1}\right]=M\left[\varepsilon_{k}\rho_{k+1}\right]=0$. As a result of the application of well-known methods [37,38], the final expression of (7), which is necessary for the creation of the algorithm for increasing the accuracy of measurement of plane surfaces, is as follows:

(9) $$M\left[\delta_{k+1}^{2}\right]={\left(1-\lambda_{k+1}\right)}^{2}\left\{M\left[\delta_{k}^{2}\right]+\sigma_{\varepsilon_{k}}^{2}\right\}+\lambda_{k+1}^{2}\sigma_{\rho_{k+1}}^{2}.$$

Determining the minimum in (6) is equivalent to the zeroing of the first derivative in the right-hand side of (9) with respect to $\lambda_{k+1}$, which leads to:

(10) $$\frac{dM\left[\delta_{k+1}^{2}\right]}{d\lambda_{k+1}}=2\left(\lambda_{k+1}-1\right)\left\{M\left[\delta_{k}^{2}\right]+\sigma_{\varepsilon_{k}}^{2}\right\}+2\lambda_{k+1}\sigma_{\rho_{k+1}}^{2}=0$$

The formula for determining the weight coefficient *λ_k+_*_1_ for each successive step of the iterative process is as follows:

(11) $$\lambda_{k+1}=\frac{M\left[\delta_{k}^{2}\right]+\sigma_{\varepsilon_{k}}^{2}}{M\left[\delta_{k}^{2}\right]+\sigma_{\varepsilon_{k}}^{2}+\sigma_{\rho_{k+1}}^{2}}$$

In order to reduce the volume of the computational operations in the algorithmic structure of the method for active compensation of the dynamic errors presented in this study, it is appropriate to provide formulae that express the relationship between $M\left[\delta_{k+1}^{2}\right]$ and $\lambda_{k+1}$. In this regard, after replacing of (11), (9) results in:

(12) $$M\left[\delta_{k+1}^{2}\right]=\sigma_{\rho_{k+1}}^{2}\frac{M\left[\delta_{k}^{2}\right]+\sigma_{\varepsilon_{k}}^{2}}{M\left[\delta_{k}^{2}\right]+\sigma_{\varepsilon_{k}}^{2}+\sigma_{\rho_{k+1}}^{2}}=\sigma_{\rho_{k+1}}^{2}\lambda_{k+1},$$

which corresponds to the formula reported in the literature [37,38] and adapted to the present specific case:

(13) $$\lambda_{k+1}=\frac{1}{\sigma_{\rho_{k+1}}^{2}}M\left[\delta_{k}^{2}\right].$$

Formula (13) is an iterative dependency for calculating the weighting factor, on the basis of which algorithms can be implemented to improve the accuracy of the measurement of flat surfaces.

## 3. An Algorithm for Increasing the Accuracy of Measurement of Flat Surfaces

**Figure 1:**
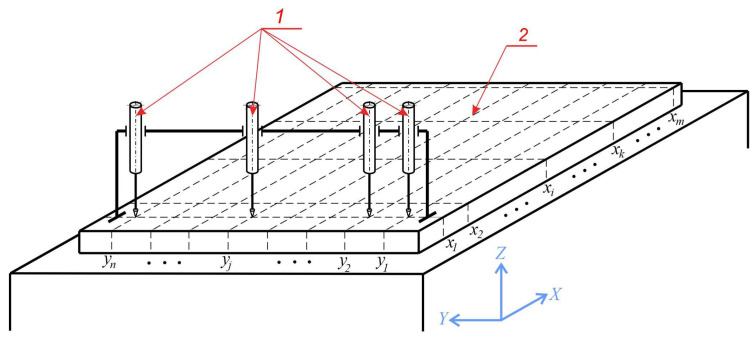
Measurement circuit: 1—linear displacement sensors; 2—flat surface.

The algorithm is developed based on the mathematical model presented above. The measurements are made based on the model shown in Figure 1.

The basic coordinate system *xyz* synchronises the metrological operations in the three-dimensional coordinate space. Linear displacement sensors 1 are placed along the *y*-axis; they measure the deviations of the flat surface 2 in the vertical direction fixed by the *z*-coordinate. The number of sensors (*j* = 1,2,…,*n*) is determined by the conditions of the specific metrological task. In operating mode, the sensors (1) are moved simultaneously in the direction set by the *x*-coordinate. Measurement data are obtained concurrently from all sensors at each sequential point *x_i_* and are recorded in matrices:

(14) $$Q=\left[\begin{array}{cccccc} q_{11} & q_{12} & \cdots & q_{1j} & \cdots & q_{1n}\\ q_{21} & q_{22} & \cdots & q_{2j} & \cdots & q_{2n}\\ \vdots & \vdots & \ddots & \vdots & \ddots & \vdots \\ q_{i1} & q_{i2} & \cdots & q_{ij} & \cdots & q_{in}\\ \vdots & \vdots & \ddots & \vdots & \ddots & \vdots \\ q_{k1} & q_{k2} & \cdots & q_{kj} & \cdots & q_{kn}\\ \vdots & \vdots & \ddots & \vdots & \ddots & \vdots \\ q_{m1} & q_{m2} & \cdots & q_{mj} & \cdots & q_{mn} \end{array}\right]$$

**Figure 2:**
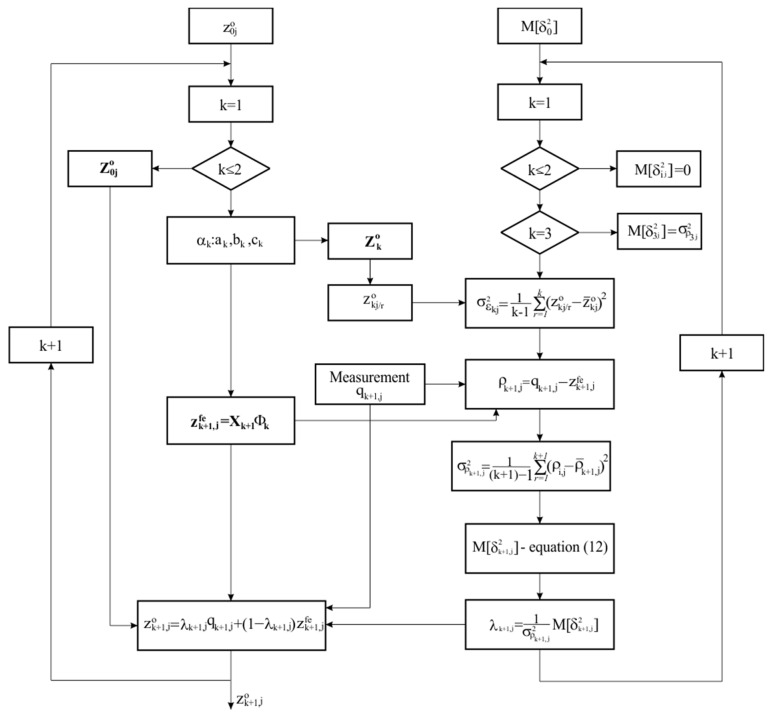
Block chart of the algorithm.

Figure 2 shows the chart that illustrates how the iterative algorithm works. The algorithm works in “predictor-corrector” mode, according to which the iterative process is divided into two main cycles: a cycle for predicting the value of the measurand in the next measurement step and a cycle for determining the value of the weighting factor through which an adjustment is made depending on the adopted optimality criterion.

**Figure 3:**
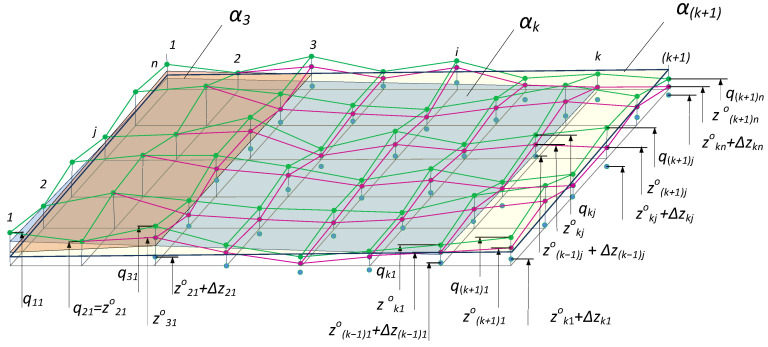
Geometric interpretation of the operation of the algorithm.

The operation of the algorithm in the first cycle is based on the theoretical model of the measured surface, which, in this case, is identical to the model of the geometric flat surface. Let the values of the optimal estimates of the measurand for each position of the sensors 1 be obtained at the point *k* of the coordinate *x*, i.e., $z_{k,j}^{0}=\left[\begin{array}{ccc} z_{k1}^{0} & z_{k2}^{0} & \cdots \end{array}\;\begin{array}{ccc} z_{kj}^{0} & \cdots & z_{kn}^{0} \end{array}\right]$. This makes it possible to calculate the coefficients $a_{k}$, $b_{k}$, and $c_{k}$ of the new reference flat surface $\alpha_{k}:a_{k}X+b_{k}Y+c_{k}=Z$ (Figure 3).

The coefficients $a_{k}$, $b_{k}$, and $c_{k}$ are calculated by the method of least squares based on the data from the vector $z_{k,j}^{0}$ and the values of the elements from all vectors preceding the point *k* and defining the flat surface $\alpha_{k+1}$, which is defined in the *k*−1 iteration. Data containing the optimal estimates of the measurand for the *k-th* iteration are recorded in the matrix

(15) $$Z_{k}^{0}=\left[\begin{array}{cccccc} z_{11}^{0} & z_{12}^{0} & \cdots & z_{1j}^{0} & \cdots & z_{1n}^{0}\\ z_{21}^{0} & z_{22}^{0} & \cdots & z_{2j}^{0} & \cdots & z_{2n}^{0}\\ \vdots & \vdots & \ddots & \vdots & \ddots & \vdots \\ z_{i1}^{0} & z_{i2}^{0} & \cdots & z_{ij}^{0} & \cdots & z_{in}^{0}\\ \vdots & \vdots & \ddots & \vdots & \ddots & \vdots \\ z_{k1}^{0} & z_{k2}^{0} & \cdots & z_{kj}^{0} & \cdots & z_{kn}^{0} \end{array}\right]$$

Each of the elements in (15) is calculated by the matrix product:

(16) $$z_{ij/k}^{0}=\left[\begin{array}{ccc} a_{k} & b_{k} & c_{k} \end{array}\right]\left[\begin{array}{c} X\\ Y\\ 1 \end{array}\right]$$

where *X* = *x_i_* (*i* = 1,2,…,*m*), *Y* = *y_j_* (*j* = 1,2,…,*n*).

The estimate for the next iteration is calculated based on the theoretical model of the flat surface $\alpha_{k}$, i.e.:

(17) $$z_{k}^{0}+\Delta z_{k}=z_{k+1,j}^{fe}=X_{k+1}\Phi_{k}=\left[\begin{array}{ccc} x_{k+1} & y_{1} & 1\\ \vdots & \vdots & \vdots \\ x_{k+1} & y_{j} & 1\\ \vdots & \vdots & \vdots \\ x_{k+1} & y_{n} & 1 \end{array}\right]\left[\begin{array}{c} a_{k}\\ b_{k}\\ c_{k} \end{array}\right]$$

The matrix $z_{k+1,j}^{fe}$ defines an “n” number of equations

(18) $$z_{k+1,1}^{fe}=a_{k}x_{k+1}+b_{k}y_{1}+c_{k}\;\vdots \;z_{k+1,j}^{fe}=a_{k}x_{k+1}+b_{k}y_{j}+c_{k}\;\vdots \;z_{k+1,n}^{fe}=a_{k}x_{k+1}+b_{k}y_{n}+c_{k}$$

on the basis of which the estimates for the iteration in the step *k +* 1 are calculated.

The basic element around which the work in the second cycle of the algorithm is synchronized is the set of models that determine the characteristics of the errors *ε* and *ρ*. The statistical characteristics of the model error *ε* can be determined by a probability vector of the parameters defining the deviations of the flat surfaces $\alpha_{r}:a_{r}X+b_{r}Y+c_{r}=Z$ from the nominal flat surface. The flat surfaces $\alpha_{r},r=1,2,\ldots ,k$ are the reference flat surfaces whose parameters are determined at each step of the iterative process. Then the error of the model *ε* will be a multivariate random variable with $M\left[\varepsilon \right]=0$ and random vector:

(19) $$\varepsilon ={\left[\begin{array}{ccc} z_{\alpha_{1}} & z_{\alpha_{2}} & \cdots \end{array}\;\;\;\;\begin{array}{ccc} z_{\alpha_{r}} & \cdots & z_{\alpha_{k}} \end{array}\right]}^{T}$$

whose elements $z_{\alpha_{r}}$ are vectors, related to the statistical interpretation of the flat surfaces $\alpha_{r},r=1,2,\ldots ,k$. On the other hand, finding a covariance matrix that has statistically interpretable parameterization and is not bound by constraints (with the exception of $M\left[\varepsilon \right]=0$) is a complex task involving a large volume of computational operations. Therefore, in this algorithm the statistical characteristics of the error *ε* are determined on the basis of quantities that include in their genesis both the covariance between the statistical parameters of the possible variants of the model and the possibility to adequately approximate the probability distribution of the error *ε* according to statistically identifiable models. As one can see from (19), the random vector *ε* is defined in the probability space $\left(Z^{0},Z_{ij}^{\varepsilon },P\right)$. The elements in the probability space include: the set $Z^{0}$, consisting of the optimal estimates $Z_{ij/r}^{\varepsilon }$, lying in each of the flat surfaces $z_{\alpha }$, defined for each coordinate point $x_{i}y_{j}$, the subset $Z_{ij}^{\varepsilon }$, consisting of random variables formed by the projection of the random vector *ε* in the direction of the measuring coordinate for each of the measuring points of the object; the probability P. For example, at a point with the coordinates $x_{k}y_{j}$ the corresponding random variable $z_{kj}^{\varepsilon }=\left(z_{kj/1}^{0},\;z_{kj/2}^{0},\;\ldots ,\;z_{kj/r}^{0},\;\ldots ,\;z_{kj/k}^{0}\right)$, part of the subset $Z_{ij}^{\varepsilon }$, has a statistical distribution that can be approximated based on the values obtained as optimal estimates for the specific coordinate point at each step of the iterative procedure. This way, the values $z_{kj/k}^{0}$ lying on the respective reference flat surfaces $\alpha_{r}$ form the random variable $z_{kj}^{\varepsilon }$, which has a mathematical expectation $M\left[z_{kj}^{0}\right]={\bar{z}}_{kj}^{0}$ and unbiased sample variance estimator:

(20) $$\sigma_{\varepsilon_{kj}}^{2}=\frac{1}{k-1}\overset{k}{\underset{r=1}{\sum }}{\left(z_{kj/r}^{0}-{\bar{z}}_{kj}^{0}\right)}^{2}$$

In practice, the mathematical expectations of the quantities $z_{ij}^{\varepsilon }$ forming the subset $Z_{ij}^{\varepsilon }$ can take values other than zero, despite the constraint $M\left[\varepsilon \right]=0$. Based on (20), the values of the variances are obtained in the *k-th* step of the algorithm:

(21) $$\sigma_{\varepsilon_{kj}}^{2}=\left(\sigma_{\varepsilon_{k1}}^{2},\sigma_{\varepsilon_{k2}}^{2},\ldots ,\sigma_{\varepsilon_{kj}}^{2},\ldots ,\sigma_{\varepsilon_{kn}}^{2}\right)$$

The values of the variances characterize the model error ε in each position of the sensors 1 (Figure 1)

In order to increase the sensitivity of the algorithm to the specifics of the measurement error ρ, the probability characteristics of this error are determined on the basis of the statistical data obtained from measurement done for each of the sensors. For example, the error of the *j-th* sensor in the *k* + 1*-th* iteration will be:

(22) $$\rho_{k+1,j}=q_{k+1,j}-z_{k+1,j}^{fe}$$

where $q_{k+1,j}$ is the measured value from the *j-th* sensor in the *k*+1-*th* iteration; $z_{k+1,j}^{fe}$ is the estimate at a point with coordinates $x_{k}+1y_{j}$.

The number of values *k*+1 which form the quantity $\rho_{ij}^{k+1}=\left(\rho_{1,j},\rho_{2,j},\ldots ,\rho_{i,j},\ldots ,\rho_{k+1,j}\right),\;i=1,2,\ldots ,k+1$ is determined on the basis of Equation (22). The quantity is a random variable with variance:

(23) $$\sigma_{\rho_{k+1,j}}^{2}=\frac{1}{\left(k+1\right)-1}\overset{k+1}{\underset{i=1}{\sum }}{\left(\rho_{i,j}-{\bar{\rho }}_{k+1,j}\right)}^{2}$$

where ${\bar{\rho }}_{k+1,j}=M\left[\rho_{ij}^{k+1}\right]$ is the mathematical expectation of the quantity $\rho_{ij}^{k+1}$.

The value of the variance $\sigma_{\rho_{ij}}^{2}$ is updated at each iteration step; the index *i* determines the sequence number of the iteration. The actual value of $\sigma_{\rho_{ij}}^{2}$ is calculated in the *k*+1*-st* step on the basis of the updated measurement data; “*n*” number of variances $\sigma_{\rho_{k+1,1}}^{2},\sigma_{\rho_{k+1,2}}^{2},\ldots ,\sigma_{\rho_{k+1,j}}^{2},\ldots ,\sigma_{\rho_{k+1,n}}^{2}$ are determined; the variances define the extent of the error ρ for each of the sensors used, respectively.

The two cycles of the algorithm work separately to reduce the computational burden; the data from the two matrices shown in (14) and (15) are used. The initial values required to start the work in the first cycle are determined by the matrix:

(24) $$Z_{0j}^{0}=\left[\begin{array}{cccccccccccccc} z_{11}^{0} & = & q_{11} & z_{12}^{0} & = & q_{12} & \cdots & z_{1j}^{0} & = & q_{1j} & \cdots & z_{1n}^{0} & = & q_{1n}\\ z_{21}^{0} & = & q_{21} & z_{22}^{0} & = & q_{22} & \cdots & z_{2j}^{0} & = & q_{2j} & \cdots & z_{2n}^{0} & = & q_{2n} \end{array}\right]$$

The mathematical expectation in the first step of the algorithm in the second cycle, i.e., for iterations 1 and 2 is $M\left[\delta_{ij}^{2}\right]=0;\left(i=1,2;j=1,\ldots ,n\right)$. The actual work in this cycle starts from the third iteration in which the mean squared value of the error δ is calculated on the basis of the following equation:

(25) $$M\left[\delta_{3j}^{2}\right]=\sigma_{\rho_{3j}}^{2}\;\sigma_{\rho_{3j}}^{2}=\frac{1}{6}\rho_{3j}^{2}\;\;\;\;\rho_{3j}=q_{3j}-z_{3j}^{fe}$$

The Equation (25) set the initial values for starting the operation in the second cycle. In the next iterations of this cycle, the mean squared error is calculated based on (12).

## 4. Experimental Studies and Results

The practical application of the mathematical model and the algorithm presented above is related to the development of methods for accurate measuring of deviations from flatness, straightness, and parallelism. The model and the algorithm can be easily adapted to perform measurements not only of the deviations mentioned above, but also of other deviations from form and location. All this will be proposed for discussion in other scientific papers, as the purpose of this paper is to present the basic mathematical apparatus and to prove its effectiveness in measuring linear quantities in conditions similar to working conditions.

**Figure 4:**
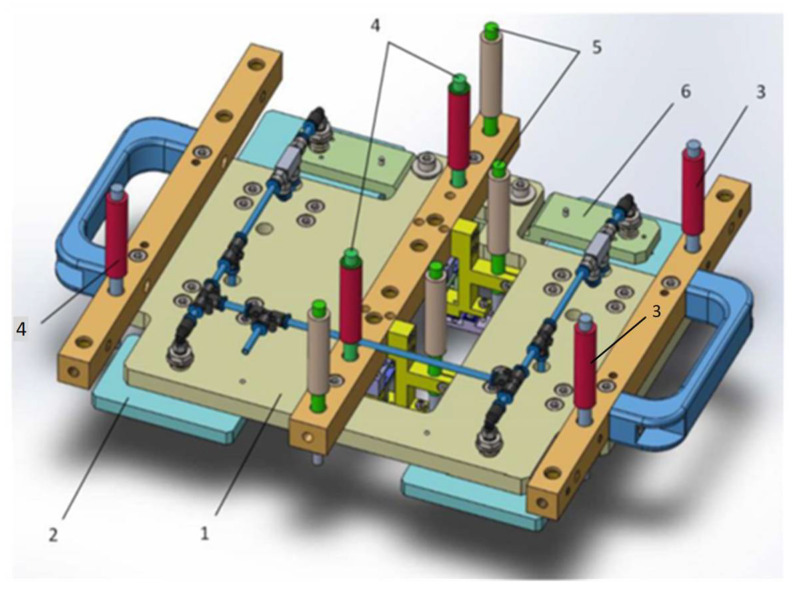
3D model of the measurement system: 1—bearing support; 2—aerostatic supports; 3—linear displacement sensors; 4, 5—corrective sensors; 6—rocker arm.

**Figure 5:**
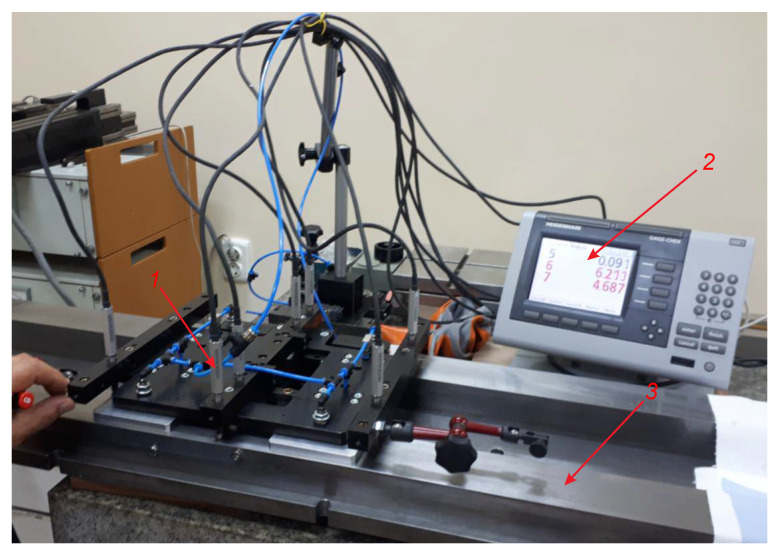
Photographs of the measuring system: 1—sensors ST1288 Heidenhein; 2—multi-channel readout devices GAGE-CH ECK ND2108G; 3—measured part.

A special measuring system for conducting experimental investigations has been built. Its 3-D model is shown in Figure 4, and Figure 5 shows its physical realization. The system is designed so that it is possible to conduct experimental investigations that support not only the objectives and tasks of the present study, but also those that relate to the measurement of deviations from flatness, straightness, and parallelism.

**Figure 6:**
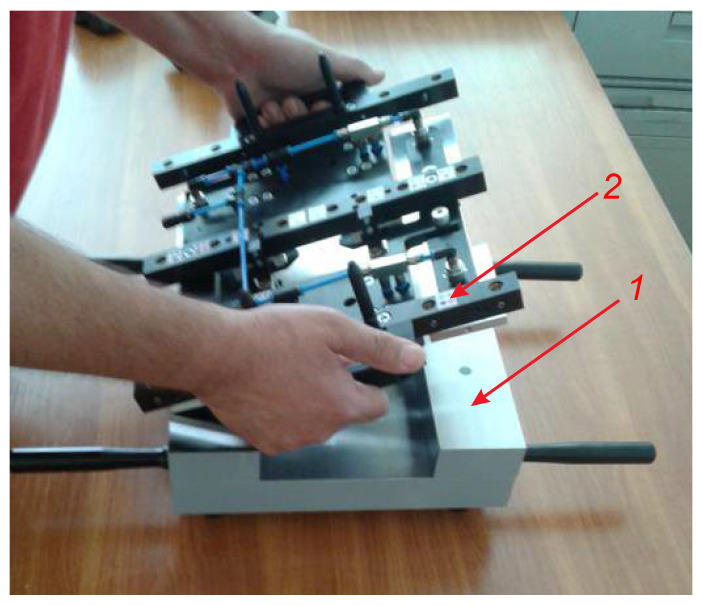
Photographs of the reference part and the measuring system: 1—reference part; 2—measuring system.

In order to recreate the performance characteristics in real conditions more accurately, aerostatic supports 2 are installed to the measuring system; they are a characteristic component of modern measurement and technological equipment (Figure 4). The linear displacement sensors 3 are fixed on the bearing support 1; the sensors are arranged according to the diagram in Figure 1. The sensors used are of the type ST1288 HEIDENHAIN with a division value of i = 0.001 mm and are connected to the multi-channel reading device GAGE-CH ECK ND2108G. According to the algorithm proposed in this paper, the data from the readout devices is fed for processing to a computer. The experimental equipment of the system also includes the reference part (position 1, Figure 6) and the measured part (position 3, Figure 5). The latter is 1900 mm long and is used as a geometric object for experimental research.

Investigations were conducted with the help of a hexapod, which provides a variety of possibilities for simulating mechanical effects that are actually present in real working conditions. The hexapod allows generation of motions along three angular and three linear coordinates with a wide range of displacement and a wide range of variation in kinematic parameters. The simulation stand can operate in the mode of generation of harmonic, poly-harmonic, or random oscillations, as well as in the mode of setting pre-recorded effects in the working environment. For the simulation of the mechanical effects in the real working environment for the specific studies, a hexapod type “Mistral” of the Symetrie company was used. These types of hexapods are known for their high accuracy and stability, which makes them suitable for precise measurements and tests. The hexapod provides the ability to generate movements along three linear coordinates (x, y, z) with maximum deviations of ±250 mm and angular deviations of up to ±30°. These parameters allow the formation of a variety of dynamic conditions that accurately simulate the real impacts to which the measuring equipment may be subjected. In order to recreate the characteristics of work in real conditions more accurately, aerostatic supports are installed in the measuring system, which are a characteristic element of the composition of modern measuring machines and technological equipment (Figure 4). Before starting the measurements, the sensors were calibrated against a reference part using standard procedures for resetting the readings. Laboratory conditions are strictly controlled, including the temperature regime, to minimize thermal deformations and mechanical vibrations that could affect the accuracy of the measurements.

In the experimental setup used in the present study, various potential sources of error that could affect the accuracy and reliability of the results were identified and minimized. One of the main factors leading to errors is the temperature fluctuations, which can cause temperature expansions of the measured parts and the measuring system itself. To minimize these effects, studies are conducted in a controlled, constant-temperature laboratory environment using materials with low thermal expansion. Mechanical vibrations are another significant source of error. To reduce their influence, the measuring system is mounted on aerostatic supports that provide stability and isolation from external vibrations. Electromagnetic interference can also be a source of errors. Although no significant problems were identified in the present experiments, the use of shielding and additional isolation of the electronic components is envisaged for future research if an influence of electromagnetic fields on the accuracy of the system is found. The accuracy of all measuring instruments is maintained by conducting regular calibrations. In order to increase the accuracy of the measurements, an intermediate calibration of the sensors was carried out when the temperature changed or other conditions occurred that could affect the measurements.

**Figure 7:**
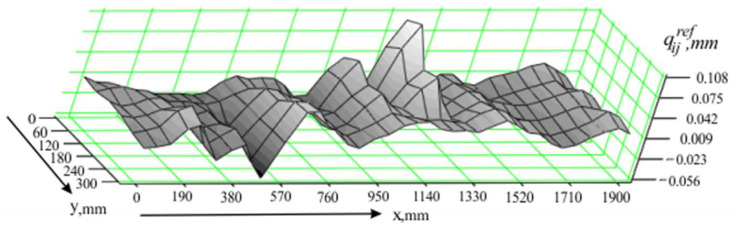
Three-dimensional model of the deviations $q_{ij}^{ref}$.

Data obtained when measuring the flatness of part 3 (Figure 5), in conditions in which there are no external effects, are used as a reference base in conducted investigations. Before starting the measurements, the readings of the sensors of the measuring system are reset according to the flatness of the reference part (Figure 6). Thus, when measuring part 3 (Figure 5), the deviations $q_{ij}^{ref}$, (*i* = 1,2,…,*m*; *j* = 1,2,…,*n*) from the zero readings of the sensors at each fixed point $x_{i}y_{j}$ of the measured coordinate sequence are obtained. The measurement data are recorded in the matrix $Q_{ref}$ with dimensions *m*×*n*, which is structurally analogous to the matrix (14). The graph of the deviations $q_{ij}^{ref}$, presented in the three-dimensional model, is shown in Figure 7.

**Figure 8:**
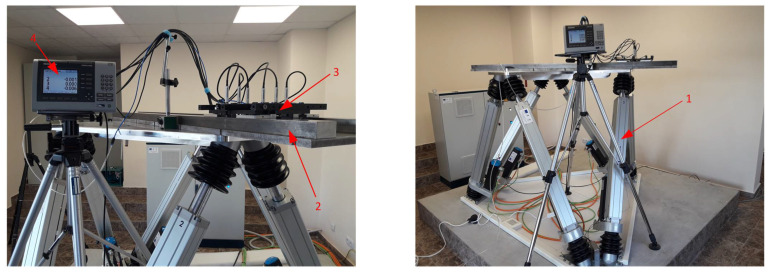
Photographs from experimental studies: 1—hexapod; 2—measured part; 3—measurement system; 4—multi-channel readout devices.

The investigations were conducted on the basis of four types of hexapod simulations, and photographs from the experiments are shown in Figure 8. In the first type of simulations, the vector of mechanical effects generated by the hexapod is pointed in the vertical direction (Figure 1). The studies simulate the dynamics of the working environment, as the signal controlling the movement of the hexapod are recorded in real production conditions. In the second mode, the simulations were performed with mechanical impacts along the x-coordinate, simulating transverse vibrations. The third mode includes actions along the y-coordinate, which simulates longitudinal vibrations. The fourth and final mode covers a combination of impacts in all three directions (x, y, z), representing complex dynamic loading. Each of these modes was studied to evaluate the performance of the proposed methods and algorithms in different operating conditions.

**Figure 9:**
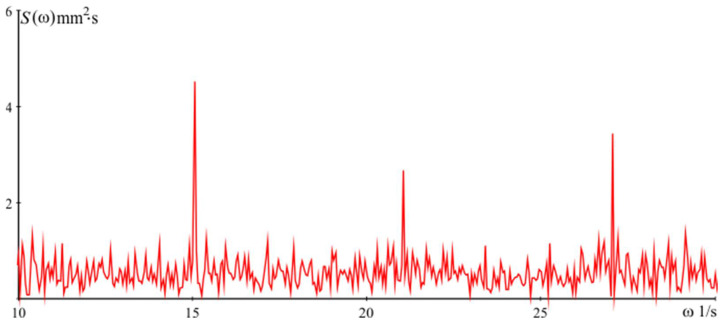
Power spectral density of simulations in vertical direction.

**Figure 10:**
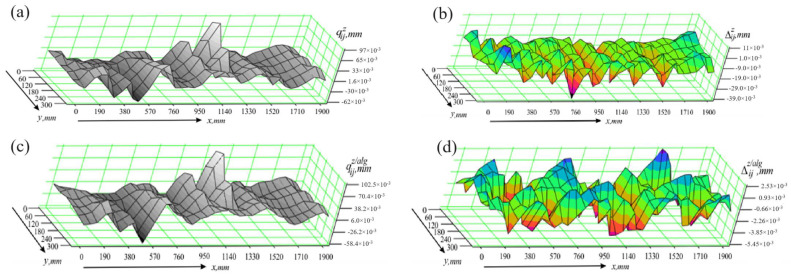
Results of studies in the presence of vertical effects; (**a**) measurement results obtained without using the algorithm module; (**b**) measurement errors occurring when the algorithm module is not used; (**c**) measurement results obtained using the algorithm module; (**d**) measurement errors occurring when the algorithm module is used.

Power spectral density of the signal $S\left(\omega \right)$ used for the simulations of dynamics in the vertical direction is presented in Figure 9. In the presented graph of the spectral density of the vibration signal recorded in real working conditions, three significant peaks of frequencies are noticeable. These peaks were observed at frequencies of 15 Hz, 21 Hz, and 27 Hz. The peak at 15 Hz is due to the main vibrations generated by the operation of the heavy equipment in the workshop. The frequencies of 21 Hz and 27 Hz can be explained by harmonic and resonance effects associated with the mechanical systems and motors used in the manufacturing process. These peaks are typical of industrial environments and show the typical vibration profiles that can affect measurements on flat surfaces. In order to evaluate the effectiveness of the algorithm operation, the measurements of the part are performed both with and without the algorithm operation module. The measurement results obtained without the module $q_{ij}^{z}$ (i.e., the algorithm is not involved in the measurement procedure) are shown in Figure 10a.

To show the range of values the measurement error can have in this mode of measurement, the following differences are defined:

(26) $$\Delta_{ij}^{z}=q_{ij}^{z}-q_{ij}^{ref}$$

where $q_{ij}^{z}$ are the results from measuring the flat surface of the part at each coordinate point $x_{i}y_{j}$, obtained in the presence of effects in vertical direction.

The analysis of measurement accuracy in this measurement mode is performed on the basis of two statistical characteristics—the maximum value $\Delta_{max}^{z}$ of the differences $\Delta_{ij}^{z}$ and their standard deviation $\sigma_{\Delta }^{z}$, which can be defined by:

(27) $$\sigma_{\Delta }^{z}=\sqrt{\frac{1}{mn-1}\overset{m}{\underset{i=1}{\sum }}\overset{n}{\underset{j=1}{\sum }}{\left(\Delta_{ij}^{z}-{\bar{\Delta }}^{z}\right)}^{2}}$$

where ${\bar{\Delta }}^{z}$ is the arithmetic mean of the differences $\Delta_{ij}^{z}$.

The differences $\Delta_{ij}^{z}$ are presented graphically in Figure 10b, and the values of the statistical characteristics obtained in the presence of vertical effects and without the algorithm operation module are $\Delta_{max}^{z}=39\;\mu m$, $\sigma_{\Delta }^{z}=6.72\;\mu m$. One can see that the measuring process is very sensitive to mechanical effects in the vertical direction. Errors, which can reach values several times higher than the error of the measuring instruments, occur. In contrast, the proposed algorithm shows efficiency in terms of measurement accuracy. The results from measuring the part $q_{ij}^{z/alg}$ employing the operation algorithm module are shown in Figure 10c. These results are close to the values of the deviations $q_{ij}^{ref}$ obtained when measuring the part in static conditions. This can be clearly seen from Figure 10d, which shows the graphical representation of the differences $\Delta_{ij}^{z/alg}=q_{ij}^{z/alg}-q_{ij}^{ref}$, as well as from the values of the statistical characteristics $\Delta_{max}^{z/alg}=5.4\;\mu m$ and $\sigma_{\Delta }^{z/alg}=1.73\;\mu m$.

**Figure 11:**
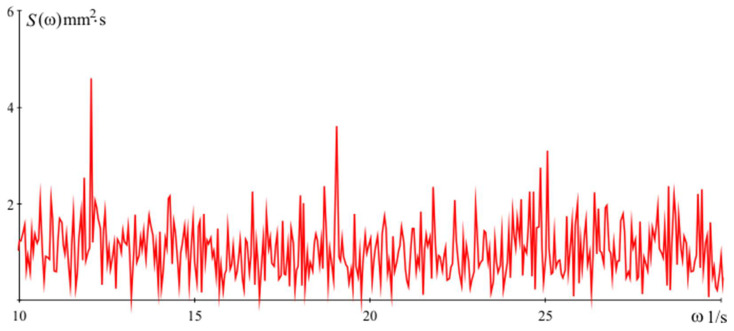
Power spectral density of simulations along the x axis.

The results from the experimental studies carried out with simulations of mechanical effects in the other two directions confirm the effectiveness of the algorithm in increasing measurement accuracy. The operation of the algorithm in the presence of mechanical effects in the direction of the axis *x* (Figure 1) was studied by simulations whose signal is recorded in real working conditions with power spectral density S(ω), shown in Figure 11.

**Figure 12:**
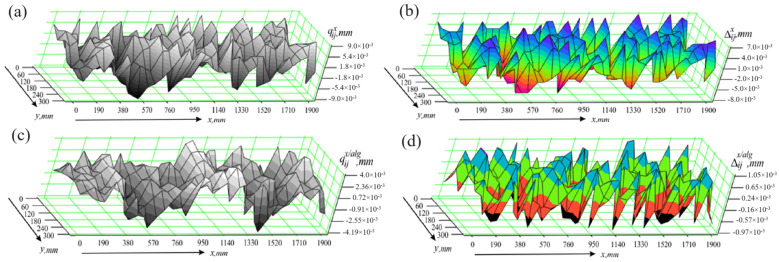
Results of studies in the presence of effects along the x axis; (**a**) measurement results obtained without using the algorithm module; (**b**) measurement errors occurring when the algorithm module is not used; (**c**) measurement results obtained using the algorithm module; (**d**) measurement errors occurring when the algorithm module is used.

The values of the deviations $q_{ij}^{x}$ obtained when measuring the flat surface of the part without the algorithm operation module are graphically presented in Figure 12a. In this way of conducting experiments, the differences $\Delta_{ij}^{x}=q_{ij}^{x}-q_{ij}^{ref}$ can reach significant values, as seen in Figure 12b. The corresponding statistical characteristics are $\Delta_{max}^{x}=8\;\mu m$ and $\sigma_{\Delta }^{x}=3.82\;\mu m$. The use of the algorithm leads to an increase in accuracy, as seen from both the results of the measurement of the part $q_{ij}^{x/alg}$, presented graphically in Figure 12c, and the calculated differences $\Delta_{ij}^{x/alg}=q_{ij}^{x/alg}-q_{ij}^{ref}$, shown in Figure 12d. In this case, the statistical characteristics have the following numerical values: $\Delta_{max}^{x/alg}=1.05\;\mu m$; $\sigma_{\Delta }^{x/alg}=0.584\;\mu m$.

**Figure 13:**
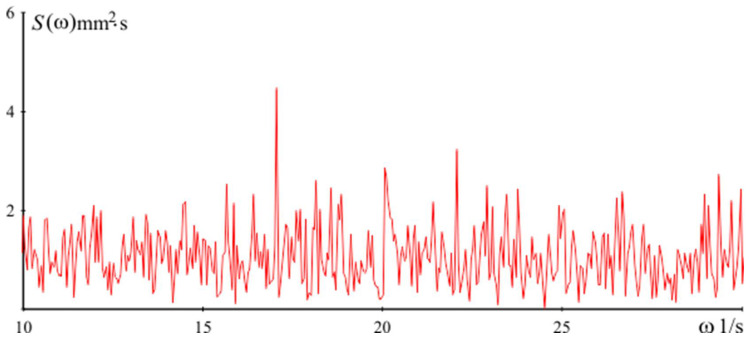
Power spectral density of simulations along the y axis.

Similar results were obtained when studying the efficiency of the algorithm in the presence of mechanical effects in the *y*-axis direction (Figure 1). The signal controlling the hexapod simulations has power spectral density S(Δ), shown in Figure 13.

**Figure 14:**
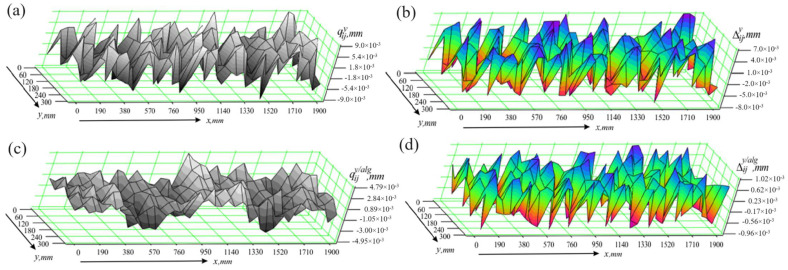
Results of studies in the presence of effects along the y axis; (**a**) measurement results obtained without using the algorithm module; (**b**) measurement errors occurring when the algorithm module is not used; (**c**) measurement results obtained using the algorithm module; (**d**) measurement errors occurring when the algorithm module is used.

Figure 14 shows the results of the study. The influence of the external effects on the measurement accuracy is significant when the processing module of the algorithm is not included. As one can see from Figure 14a,b, the deviations $q_{ij}^{y}$ and the differences $\Delta_{ij}^{y}=q_{ij}^{y}-q_{ij}^{ref}$ can reach significant values, thus considerably reducing accuracy of measurement. This is also proved by the calculated values of the statistical characteristics, which in this case are as follows: $\Delta_{max}^{y}=8\;\mu m$; $\sigma_{\Delta }^{y}=4.277\;\mu m$. Measurement accuracy is improved significantly when the algorithm proposed in this study is included in the measurement process. Figure 14c shows the results of the measurement of the part $q_{ij}^{y/alg}$; the differences from the reference readings $\Delta_{ij}^{y/alg}=q_{ij}^{y/alg}-q_{ij}^{ref}$ shown in Figure 14d are significantly smaller than the differences $\Delta_{ij}^{x}$ at the respective coordinate points $x_{i}y_{j}$. The statistical characteristics in this mode of operation have the values $\Delta_{max}^{y/alg}=1.02\;\mu m$; $\sigma_{\Delta }^{y/alg}=0.595\;\mu m$, which compared to $\Delta_{max}^{y}$ and $\sigma_{\Delta }^{y}$ prove the efficiency of the algorithm.

The presented results of experimental studies show that the inertial effects significantly influence the accuracy of measuring flat surfaces. The highest values of the error occurring in the measurement process are obtained when the vector of mechanical effects is directed vertically. However, this depends on the dynamic stability of the inertial elements of the specific sensors used in the measurement process in each of the three directions. The algorithm for active compensation of dynamic errors proposed in this study performs well, regardless of the direction of inertial effects.

**Figure 15:**
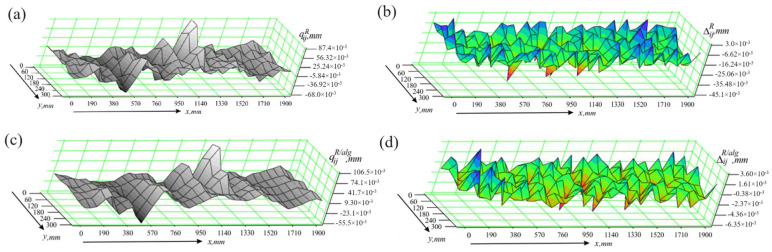
Results of studies in the presence of effects along the three axes; (**a**) measurement results obtained without using the algorithm module; (**b**) measurement errors occurring when the algorithm module is not used; (**c**) measurement results obtained using the algorithm module; (**d**) measurement errors occurring when the algorithm module is used.

This is also proved by the experimental studies, conducted by simulations of the hexapod whose vector consists of the sum of the vectors defining the composite movements along three coordinates *x*, *y,* and *z*. The movements of the hexapod along the coordinates *x*, *y,* and *z* are controlled by signals whose power spectral densities correspond to the curves presented in Figure 9, Figure 11 and Figure 13. This mode of operation has the most adverse effect on the measurement accuracy, as seen in Figure 15a,b, which show the measurement results of the part $q_{ij}^{R}$ and the calculated differences from the reference readings $\Delta_{ij}^{R}=q_{ij}^{R}-q_{ij}^{ref}$. Despite the fact that the measurement errors occurring in this mode of simulations have comparatively high values, the algorithm significantly improves accuracy and reduces measurement errors to values comparable to those in the previous three modes. The numerical values of the measurement results of the part $q_{ij}^{R/alg}$ and their corresponding differences $\Delta_{ij}^{R/alg}=q_{ij}^{R/alg}-q_{ij}^{ref}$ are graphically presented in Figure 15c,d.

Table 1 presents comparative data on the maximum error values and their corresponding root mean square deviations when measuring flat surfaces with and without the use of the proposed algorithmic correction. The data shown illustrate the effectiveness of the dynamic error correction method in four different modes of external impact simulation.

## 5. Conclusions

A mathematical model for eliminating the influence of external effects on the accuracy of measurement of flat surfaces has been proposed. The core concept of the model is an active compensation of dynamic errors based on the theoretical model of the measured surface for obtaining the required estimate, which can be largely corrected based on the analysis of the current measurements in the ordered coordinate sequence. Based on this model, an algorithm for real-time data processing has been developed. The algorithm works in “predictor-corrector” mode and is structured in two cycles. The operation of the algorithm in the first cycle is based on the theoretical model of the measured surface; in the second cycle, a weighting factor enabling the determination of the estimate in the respective step of the measurement procedure is calculated. The accuracy of the algorithm is ensured by obtaining the best estimate in each step of the iterative process, based on minimizing the variance of random component, where the main values are formed by the accumulated statistics of the model error and the measurement error.

This paper presents the results of conducted experimental studies. The experiments were carried out using a hexapod to generate disturbing effects recorded in real working conditions. A special measuring system, which has the basic properties of coordinate systems with respect to their dynamic characteristics, has been developed to perform experimental studies. The results of experimental studies conducted with simulations of mechanical effects in four modes of operation of the hexapod show that the influence of disturbing effects on the accuracy of measurement of flat surfaces is considerable. Errors occur that are significantly greater than the error of the sensors used in the measurement system. The algorithm proposed in this study proved effective in each of the four modes used for simulation of mechanical effects. When employing the operation module of this algorithm in the measuring procedure, measurement errors are reduced several times and reach values similar to the error value of the sensors used in the measurement system. Further research in this area can be directed to the application of the proposed noise-reduction methodology in ecological and oceanographic measurements, as described in [39].

## Figures and Tables

**Table 1:** Statistical indicators of errors in different modes of operation.

**Operating Mode**	Type of Measurement			
	No Algorithm		With Algorithm	
	Parameter		Parameter	
1st mode of operation	$\Delta_{max}^{z}$, µm	$\sigma_{\Delta }^{z},\;$µm	$\Delta_{max}^{z/alg},\;$µm	$\sigma_{\Delta }^{z/alg},\;$µm
	39	6.72	5.4	1.73
2nd mode of operation	$\Delta_{max}^{x},\;$µm	$\sigma_{\Delta }^{x},\;$µm	$\Delta_{max}^{x/alg},\;$µm	$\sigma_{\Delta }^{x/alg},\;$µm
	8	3.82	1.05	0.584
3rd mode of operation	$\Delta_{max}^{y},\;$µm	$\sigma_{\Delta }^{y},\;$µm	$\Delta_{max}^{y/alg},\;$µm	$\sigma_{\Delta }^{y/alg},\;$µm
	8	4.277	1.02	0.595
4th mode of operation	$\Delta_{max}^{R},\;$µm	$\sigma_{\Delta }^{R},\;$µm	$\Delta_{max}^{R/alg},\;$µm	$\sigma_{\Delta }^{R/alg},\;µm$
	45.1	6.87	6.35	1.91

## Data Availability

Data are available within the article.
